# Lower nanometer-scale size limit for the deformation of a metallic glass by shear transformations revealed by quantitative AFM indentation

**DOI:** 10.3762/bjnano.6.176

**Published:** 2015-08-13

**Authors:** Arnaud Caron, Roland Bennewitz

**Affiliations:** 1 Leibniz - Institute for New Materials, Campus D2.2, 66123 Saarbrücken, Germany; 2 current address: Korea University of Technology and Education, Department of Energy, Materials and Chemical Engineering, Chungcheongnam-do, 31253 Republic of Korea

**Keywords:** AFM indentation, dislocation, metallic glasses, metals, plasticity, shear transformation

## Abstract

We combine non-contact atomic force microscopy (AFM) imaging and AFM indentation in ultra-high vacuum to quantitatively and reproducibly determine the hardness and deformation mechanisms of Pt(111) and a Pt_57.5_Cu_14.7_Ni_5.3_P_22.5_ metallic glass with unprecedented spatial resolution. Our results on plastic deformation mechanisms of crystalline Pt(111) are consistent with the discrete mechanisms established for larger scales: Plasticity is mediated by dislocation gliding and no rate dependence is observed. For the metallic glass we have discovered that plastic deformation at the nanometer scale is not discrete but continuous and localized around the indenter, and does not exhibit rate dependence. This contrasts with the observation of serrated, rate-dependent flow of metallic glasses at larger scales. Our results reveal a lower size limit for metallic glasses below which shear transformation mechanisms are not activated by indentation. In the case of metallic glass, we conclude that the energy stored in the stressed volume during nanometer-scale indentation is insufficient to account for the interfacial energy of a shear band in the glassy matrix.

## Introduction

Hardness testing has been widely applied by materials scientists and mechanical engineers to assess the mechanical properties of materials and to predict their behavior during machining processes or under tribological loading for the last 150 years. Most generally hardness testing describes the resistance of a material surface to the penetration of a harder indenter and thus relates in the case of metals to the resistance to plastic flow. Different types of indenter geometries have been employed, such as spherical, conical, and pyramidal indenters. For each of these, different hardness numbers have been established, such as the Brinell hardness (*HB*), the Vickers hardness (*HV*) or the Knopp hardness (*HK*). They are calculated by dividing the applied load by the area of a remaining indent. While the experimental implementation of hardness testing is straightforward the obtained quantities are rather empirical and do not describe a fundamental property of the material. A first attempt to relate the hardness of metals to their intrinsic mechanical properties was made for a spherical indenter. The hardness was set equal to the mean contact pressure and the hardness was defined as the ratio of the maximal applied load to the projected area of the remaining indent. This quantity is also referred to as the Meyer’s hardness (*H*) and relates to the yield stress of non-work-hardening metals according to *H* ≈ 3 σ_y_ [1].

In industrial practice the projected area of the remaining indent is evaluated by optical microscopy and hardness measurements are limited to the macro-scale. With the development of depth-sensing indentation techniques such as instrumented nanoindentation, the recording of load–displacement curves has been recognized as a suitable alternative to determine the hardness of a material, even when the remaining indent is too small to be accurately imaged. The projected area is then determined from the penetration depth with the help of an indenter-area function [2]. Meanwhile the Berkovich indenter with a half-opening angle α = 65.27° is widely used for depth-sensing indentation measurements so as to match the projected area to depth characteristics of the four-sided pyramidal Vickers indenter. The preference for the Berkovich indenter arises from the accurate and reproducible processing of three sided pyramids and their ability to apply larger strains than spherical indenters. Analysis of the curvature of load–displacement curves and of the occurrence of sharp pop-ins have further been used to study mechanisms of plastic deformation such as the generation and multiplication of dislocations in crystalline metals [3] or the generation of shear bands in metallic glasses [4].

Dislocation nucleation and shear band generation are mechanisms that operate at the nanometer scale. In order to investigate the fundamental mechanisms contributing to the mechanical behavior of materials new advanced experimental techniques are required with a resolution at the level of the relevant structures. First studies of single plasticity events in nanometer scale contacts between a nanometer-sized single asperity and Au(111), (110), and (001) surfaces have been performed by means of interfacial force microscopy (IFM) [5–6]. Indentation by means of atomic force microscopes (AFM indentation) has been used to observe the nucleation and gliding of single dislocations in a KBr(100) single crystal [7] and in Cu(100) [8]. Pop-ins were observed in load–displacement curves, and the pop-in length observed in AFM-indentation was in the range of 1 Å and less. These observations demonstrate the potential of AFM indentation to detect atomistic plasticity events.

Here we present the results of AFM indentation in ultra-high vacuum to quantitatively determine hardness and the underlying fundamental mechanisms of plastic deformation of nanometer-scale contacts between an AFM-tip and a Pt(111) single crystal on the one hand, and a Pt_57.5_Cu_14.7_Ni_5.3_P_22.5_ metallic glass on the other hand. In order to investigate plasticity mechanisms at the nanometer-scale, AFM indentation experiments were performed with varying maximal loads and varying loading rates. We discuss our results with regard to dislocation activity in crystalline materials and to the recent discussion of plasticity mechanisms in metallic glasses, including the generation of shear bands and their incipient size and indentation size effects down to the structural length scale of metallic glasses [9–12].

## Experimental

The sample preparation followed a similar method as already described in [13]. The (111) surface of a platinum single crystal was prepared by several cycles of Ar sputtering and annealing at 1000 °C. This resulted in the formation of 50 to 100 nm wide atomically flat terraces. A Pt_57.5_Cu_14.7_Ni_5.3_P_22.5_ metallic glass master alloy was prepared according to [14] and subsequently melt-spun on a Cu wheel to produce 20 µm thick amorphous metallic ribbons. The amorphous structure of the Pt-based metallic glass was confirmed by X-ray diffraction (XRD) with Cu Kα radiation and differential scanning calorimetry (DSC). We observed a broad diffraction peak at 39.866 degrees of 2θ, a clear glass transition at *T*_g_ = 223 °C, and crystallization at *T*_x_ = 295 °C using a heating rate *R*_H_ = 0.67 K/s. The surface of an as-spun Pt-based metallic glass ribbon was prepared by careful Ar-sputtering for a duration of 5 min with an energy of 1 keV to remove its native oxide layer. Both Pt(111) and Pt_57.5_Cu_14.7_Ni_5.3_P_22.5_ metallic glass surfaces were characterized with Auger electron spectroscopy (AES) that confirmed the absence of surface contaminants such as C, H, S, and O. For both samples non-contact (nc)-AFM imaging was used to determine the RMS-roughness *R*_q_ over a representative area of 250 × 250 nm^2^. For atomically flat Pt(111) we found *R*_q_ = 0.372 nm, caused by atomic steps between terraces, and for Pt_57.5_Cu_14.7_Ni_5.3_P_22.5_ metallic glass we found *R*_q_ = 0.375 nm.

The nanometer-scale plastic deformation of Pt(111) and the Pt_57.5_Cu_14.7_Ni_5.3_P_22.5_ metallic glass was investigated in ultra-high vacuum by AFM indentation and subsequent nc-AFM imaging using a VT-AFM manufactured by Omicron Nanotechnology GmbH, Germany. In non-contact AFM an AFM cantilever is driven to oscillate close to a sample surface at its resonance frequency. The tip–sample distance is of the order of a few nanometers. Changes in tip–sample distance during scanning over a sample surface due to sample topography yield changes in the oscillation amplitude and in a frequency shift of the cantilever resonance. In order to measure topography both amplitude and frequency shift are tracked by a feedback loop so as to keep the cantilever oscillation in resonance [15]. For indentation and imaging we used a diamond-coated silicon single crystalline cantilever (Type: CDT-NCLR, manufactured by NanoSensors). The bending stiffness of the cantilever was calculated according to the beam geometry method [16] and was found to be *k*_n_ = 46 N/m. The sensitivity of the photo-diode was calibrated in the non-contact mode of AFM, following the method proposed in [17] and considering a conversion factor of 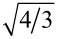 for the vibration energy of the cantilever determined from the optically measured deflection [18]. This allowed us for a precise measurement of the vertical deflection. AFM indentation measurements comprised the measurement of the cantilever deflection upon extension of the *z*-scanner of the AFM with different velocities. Given the tilt angle of the cantilever with regard to the sample surface a tilt correction was applied by moving the lateral scanner by *Z* × tan φ during a vertical scanner extension *Z*, where φ is the tilt angle [19]. In this work the extension lengths *Z* of the *z*-scanner were varied from 10 to 160 nm and the extension rate 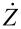 were varied from 1 to 8 nm/s.

**Figure 1:**
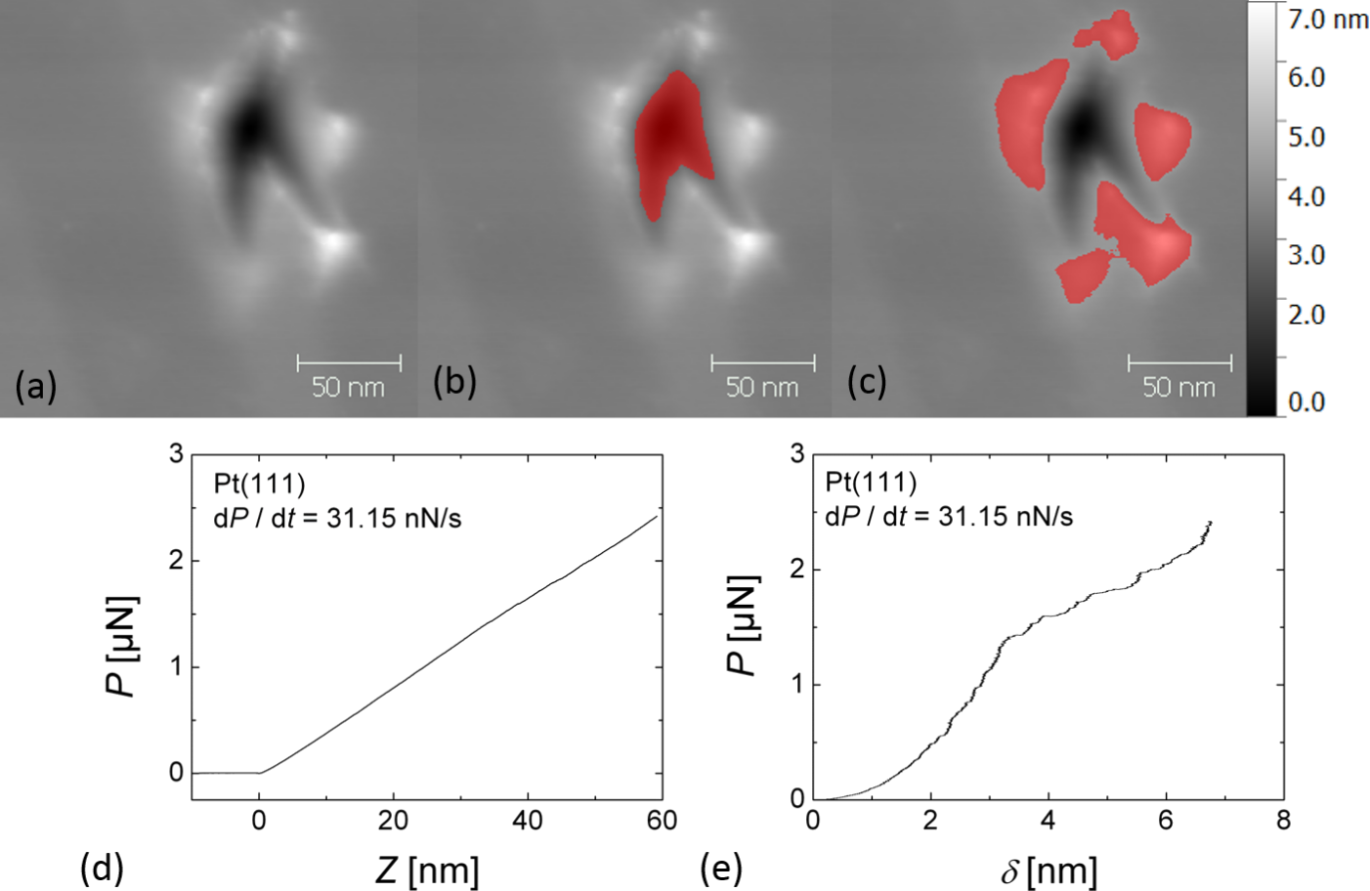
(a) Non-contact (nc)-AFM image of an AFM-indentation on Pt(111) (*z*-scale: 7 nm) and highlighted masks used to determine (b) the projected area and (c) the pile-up volume of the AFM indentation shown in (a); in (a–c) the *z*-scale is 7 nm; (d) force distance–curve recorded on Pt(111) at the indicated loading rate and (e) corresponding AFM indentation curve. As described in the text, (e) is extracted from (d) by subtracting the deflection of the cantilever.

The plastic deformation of both sample surfaces was analyzed based on nc-AFM topographical images of the indents and on the force–distance curves recorded during AFM-indentation. Figure 1a shows a typical topographical image of a Pt(111) surface after indentation with a stiff diamond-coated AFM tip. The indent was analyzed with regard to its projected area and its pile-up volume by using the indentation analysis function of the software package Gwyddion [20]. The projected area and the pile-up volume were determined by masking the area with height values below and above a reference plane (see Figure 1b,c). The reference plane was selected by setting a relative height tolerance value, so as to not include topographical features of the undeformed surface into the calculations. The values obtained for the pile-up volume and the projected area of the indent underestimate the real values by the maximal peak value of the height signal in the undeformed region of the topographical image. Due to tip-shape convolution effects the size of indents imaged by nc-AFM are underestimated while the pile-up volume is overestimated; this effect is more pronounced for smaller indents and pile-ups.

The force–distance curves were used to calculate force–penetration (*P*–δ) curves (see Figures 1d,e). For an infinitely stiff and hard sample surface the cantilever deflection *D* upon extension of the *z*-scanner is equal to the extension length *Z*, i.e., the slope d*D*/d*Z* = 1. For a compliant sample surface, an extension of the *z*-scanner also leads to a penetration of the AFM tip into the sample surface by the penetration depth δ = *Z* − *D*. While the cantilever deflection *D* is calibrated independently, the height value *Z* is subject to drift or creep effects of the piezoelectric scanner. The accuracy in δ is thus limited by piezoelectric creep of the AFM scanner. In order to minimize vertical drift, the tip position was equilibrated before each indentation by recording a slow 500 × 500 nm^2^ scan of the area to be indented by AFM. Indentation measurements were then started from the position of the scanner during nc-AFM imaging, i.e., half of the oscillation amplitude or a few nanometers above the surface.

**Figure 2:**
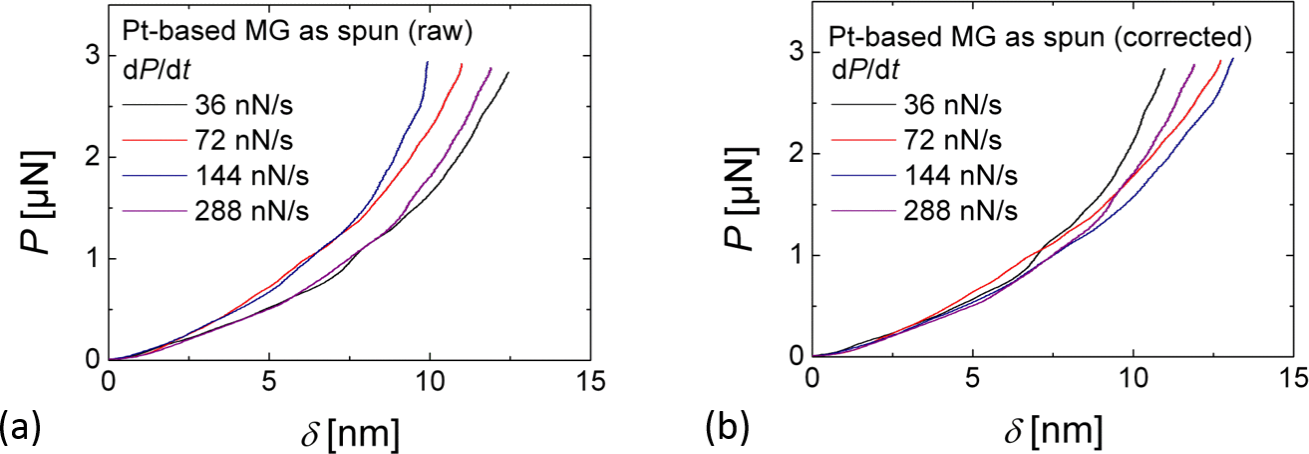
Series of rate-dependent AFM indentation curves (a) before and (b) after drift correction; the drift velocity difference with respect to the fastest measurements were Δ*v* = −19.5, 46, and 170 pm/s for d*P*/d*t* = 36, 72, and 144 nN/s, respectively.

In order to further account for piezoelectric creep effects during rate-dependent measurements a drift difference Δ*Z*_drift_ with regard to the fastest measurement was calculated according to Δ*Z*_drift_ = Δδ + Δ*D* = *v*_drift_ × *t*, where Δδ and Δ*D* are the differences in penetration depth and deflection with regard to the fastest measurement, *v*_drift_ is the drift velocity and *t* is the time. Subsequently, the penetration depth was re-evaluated according to δ = *Z* + Δ*Z*_drift_ − *D* under the assumption that *v*_drift_ = Δ*Z*_drift_/*t* remained constant during the extension of the *z*-scanner. Figure 2 shows a series of rate-dependent *P*–δ curves on Pt_57.5_Cu_14.7_Ni_5.3_P_22.5_ metallic glass before and after correction of piezoelectric drift. The absolute values for the calculated drift velocity difference vary from 19.5 to 170 pm/s. After drift correction the indentation curves coincide as expected in the low load regime that corresponds to the elastic deformation.

**Figure 3:**
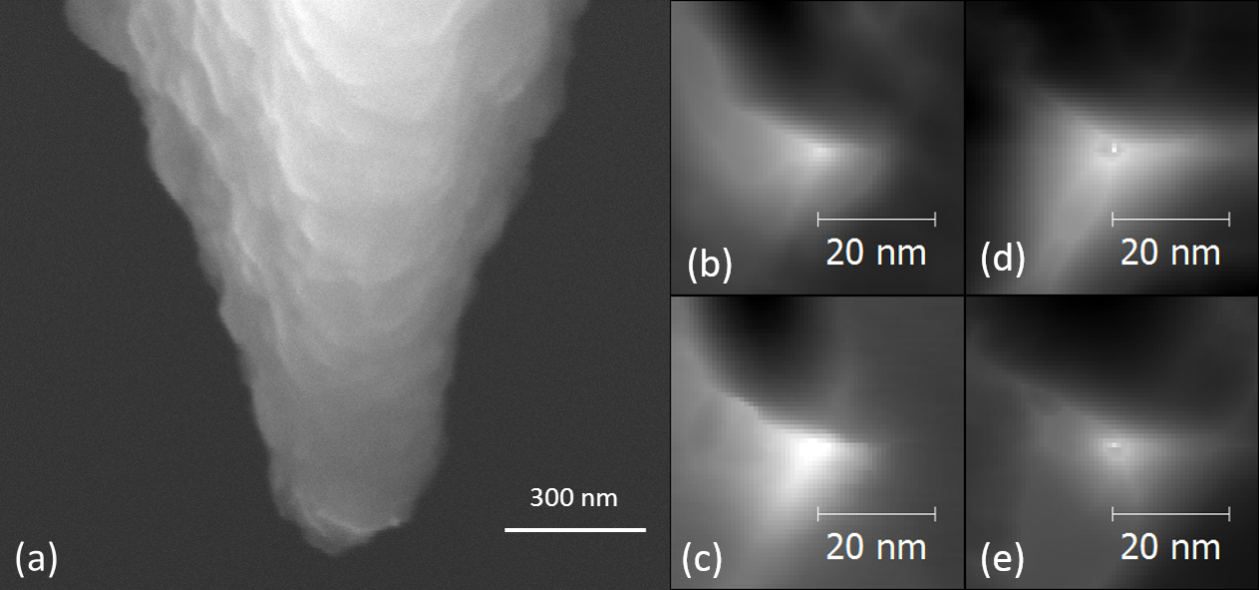
(a) SEM image of the diamond coated AFM tip used for all measurements presented in this report; tip geometry reconstructed from nc-AFM images on (b,c) Pt(111) (*z*-scale: 5 nm) and (d,e) Pt_57.5_Cu_14.7_Ni_5.3_P_22.5_ metallic glass (*z*-scale: 10 nm). The nc-AFM images used to reconstruct the tip shape in (b,d) and (c,e) were recorded several months apart.

A drawback of AFM indentation experiments is that no controlled manufacturing of the tip apex is available at the nanometer scale and one has to work with the shape of a given tip. Figure 3 shows a SEM image of the diamond-coated AFM tip after recorded after finishing all measurements presented in this report. One can recognize the granular structure of the diamond coating of the tip. From SEM images like this one it is usually not possible to identify which granule or crystallite at the tip apex was the actual indenter. However, the shape of the AFM tip relevant for indentation and imaging can be reconstructed using the tip analysis function of Gwyddion [20]. Figure 3b,c and Figure 3d,e show the shape of the AFM tip reconstructed from nc-AFM images of indents on Pt(111) and Pt_57.5_Cu_14.7_Ni_5.3_P_22.5_ metallic glass, respectively. As confirmed in all reconstructions performed over several months, the tip is a stable three-sided pyramid that we assume is the corner of a single diamond nano-grain. The angle of each side with regard to the basis of the pyramid is approx. 16°, while its half opening angle is α ≈ 74°.

## Results

**Figure 4:**
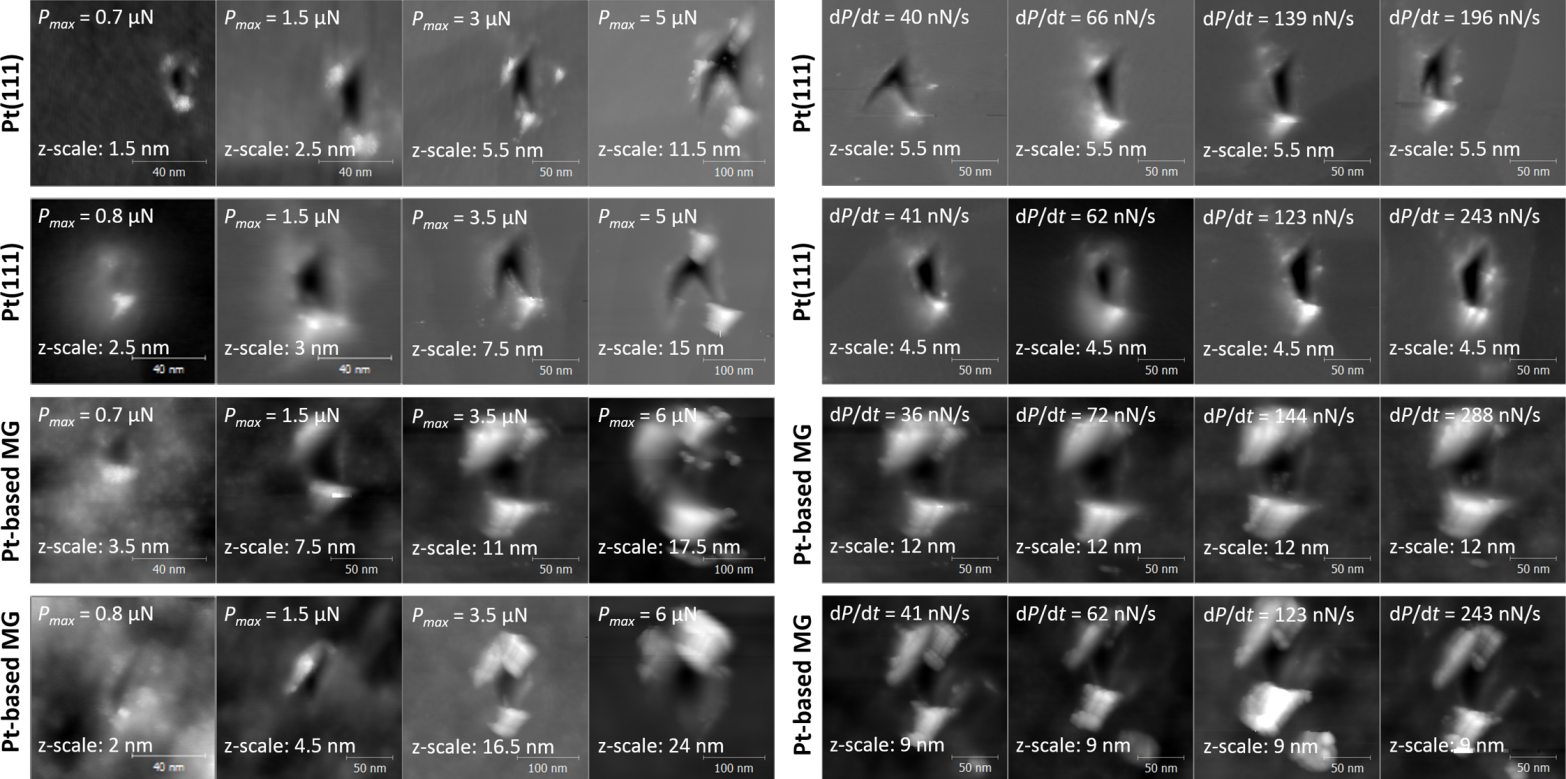
nc-AFM images of indented surfaces as a function of (left) the maximal loads *P*_max_ and of (right) the loading rate d*P*/d*t* on Pt(111) and Pt_57.5_Cu_14.7_Ni_5.3_P_22.5_ metallic glass; all AFM indentation measurements were performed with the same diamond-coated AFM tip that was analyzed in Figure 3.

Figure 4 shows series of nc-AFM images of indents on Pt(111) and Pt_57.5_Cu_14.7_Ni_5.3_P_22.5_ metallic glass for varying maximum loads and varying loading rates. The different series of this matrix have been conducted over a period of several months and on different surface preparations. Images of indents on the surface of a same sample performed at similar loads or loading rates are very similar. This similarity demonstrates the reproducibility and the stability of the tip under repeated indentation tests. For both materials remaining indents were observed only for a minimum load of *P*_max_ > 0.8 µN. For both materials the projected area of the indents increases with the load with a similar trend. Indents performed on a given sample at different loading rates with the same maximal load do not show any tendency and have similar shapes. However, the height of pile-up around indents differs significantly between Pt(111) and Pt_57.5_Cu_14.7_Ni_5.3_P_22.5_ metallic glass. Pile-ups around indents on the metallic glass are much more prominent than on Pt(111). This indicates that the plastic deformation of Pt(111) was accommodated over much longer distances than in the case of Pt_57.5_Cu_14.7_Ni_5.3_P_22.5_ metallic glass. In the case of the metallic glass plastic flow was closely confined around the indenting tip. The chevron-like shape of the indent performed on Pt(111) with *P*_max_ > 3 µN and low loading rates was never observed on Pt_57.5_Cu_14.7_Ni_5.3_P_22.5_ metallic glass. We attribute the distortion of the triangle to an anisotropic elastic relaxation of Pt(111).

**Figure 5:**
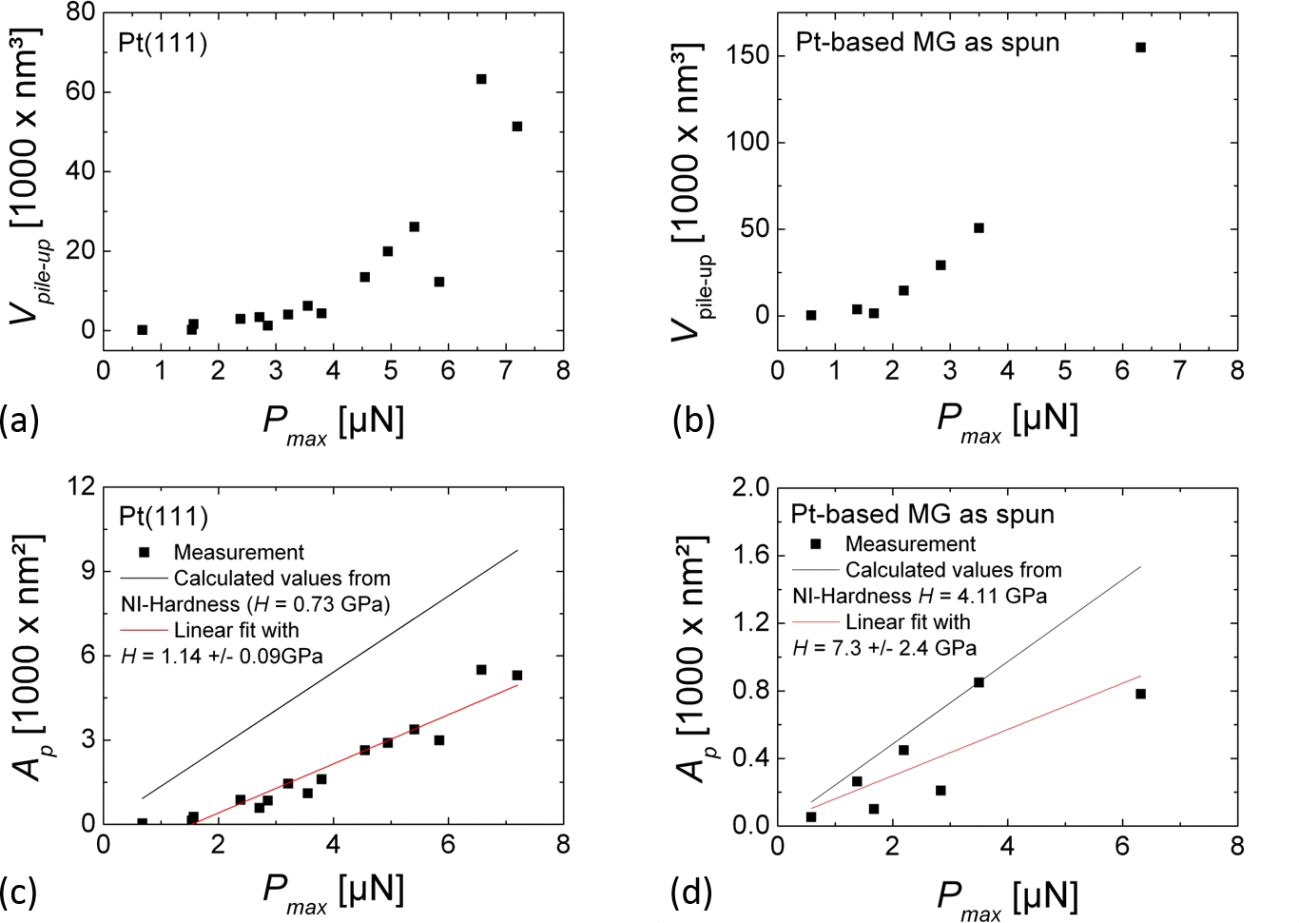
(a,b) Pile-up volume *V*_pile up_, (c,d) projected area *A*_p_ as a function of *P*_max_ for (a,c) Pt(111) and (b,d) Pt_57.5_Cu_14.7_Ni_5.3_P_22.5_ metallic glass. The data points in (c) and (d) where fitted with a linear function *A*_p_ = *P*_max_/*H* shown as a red line; for comparison the calculated values of *A*_p_ as a function of *P*_max_ are shown as a black line for hardness values measured by nanoindentation.

Figure 5 shows the load dependence of the pile-up volume *V*_pile up_ and the projected area *A*_p_ for Pt(111) and Pt_57.5_Cu_14.7_Ni_5.3_P_22.5_ metallic glass as extracted from nc-AFM images of indents. Values for *V*_pile up_ and *A*_p_ recorded in different experimental series are in very good agreement. As already observed in Figure 4, *V*_pile up_ for the metallic glass is more than a factor of two higher than for Pt(111). The measured projected area *A*_p_ of indents obtained at different maximal loads are significantly smaller for Pt_57.5_Cu_14.7_Ni_5.3_P_22.5_ metallic glass than for Pt(111). In both cases *A*_p_ is observed to increase linearly with *P*_max_. Given the fact that the pile-up volume has to be equal to the indent volume it is misleading that the values obtained for *A*_p_ on Pt(111) are larger than on Pt_57.5_Cu_14.7_Ni_5.3_P_22.5_ metallic glass, while the corresponding values for *V*_pile up_ on Pt_57.5_Cu_14.7_Ni_5.3_P_22.5_ metallic glass are significantly larger than on Pt(111). This apparent contradiction can be resolved by considering the lateral extension of plastic deformation. The nc-AFM images in Figure 4 distinctly show that in the case of the metallic glass plastic flow was concentrated around the indent. In this case *V*_pile up_ could be measured accurately. In the case of Pt(111) it appears that plastic deformation was accommodated over larger distances than considered in our image frames, resulting in an underestimation of *V*_pile up_. Furthermore the steeper increase of the pile-up volume as a function of the indentation load for Pt_57.5_Cu_14.7_Ni_5.3_P_22.5_ metallic glass compared to Pt(111) further underlines the higher localization of plastic deformation for the metallic glass.

Using the load dependence of *A*_p_, we further calculated the hardness of both sample surfaces according to d*A*_p_/d*P*_max_ = 1/*H*. Usually, the hardness is calculated as the ratio of a normal load to the projected area of a remaining indent. For smaller loads the hardness is, however, difficult to determine by this means due to the elastic offset. This explains why in this work the hardness was calculated as the slope d*A*_p_/d*P*_max_. For Pt(111) we obtained *H* = 1.14 ± 0.09 GPa; a slightly higher value than *H*_NI_ = 0.73 GPa which we found from nanoindentation measurements. For Pt_57.5_Cu_14.7_Ni_5.3_P_22.5_ metallic glass we obtained *H* = 7.3 ± 2.4 GPa, also larger than *H*_NI_ = 4.11 GPa found from nanoindentation measurements. The larger hardness values based on nc-AFM imaging are attributed to the underestimation of the projected area of indents due to tip convolution. The corresponding error for the metallic glass is larger due to the increased difficulty to accurately measure *A*_p_ from nc-AFM images. The large pile ups in metallic glass appear to partly cover the indents after indenter retraction.

**Figure 6:**
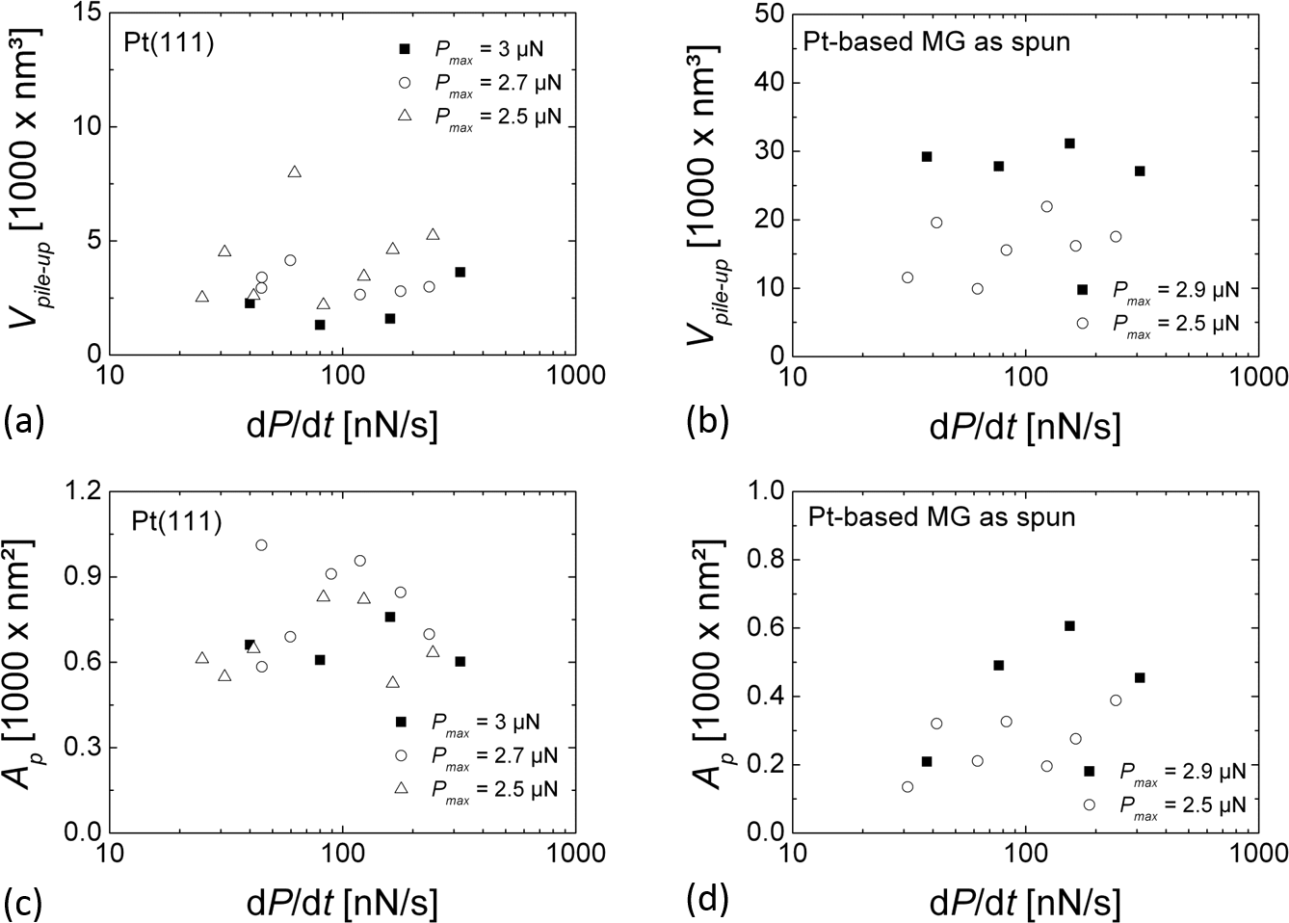
(a,b) Pile-up volume *V*_pile up_, (c,d) projected area *A*_p_ as a function of d*P*/d*t* for (a,c) Pt(111) and (b,d) Pt_57.5_Cu_14.7_Ni_5.3_P_22.5_ metallic glass, respectively.

Figure 6 shows the loading-rate dependence of the pile-up volume *V*_pile up_ and the projected area *A*_p_ for Pt(111) and Pt_57.5_Cu_14.7_Ni_5.3_P_22.5_ metallic glass as evaluated from nc-AFM images of indents from different series of indentations. For both samples *V*_pile up_ was found to be independent on the loading rate d*P*/d*t*. The pile-up volume *V*_pile up_ is again found to be significantly higher on Pt_57.5_Cu_14.7_Ni_5.3_P_22.5_ metallic glass than on Pt(111). For both samples the indent area *A*_p_ was found to be independent of the loading rate as well (see Figure 6).

**Figure 7:**
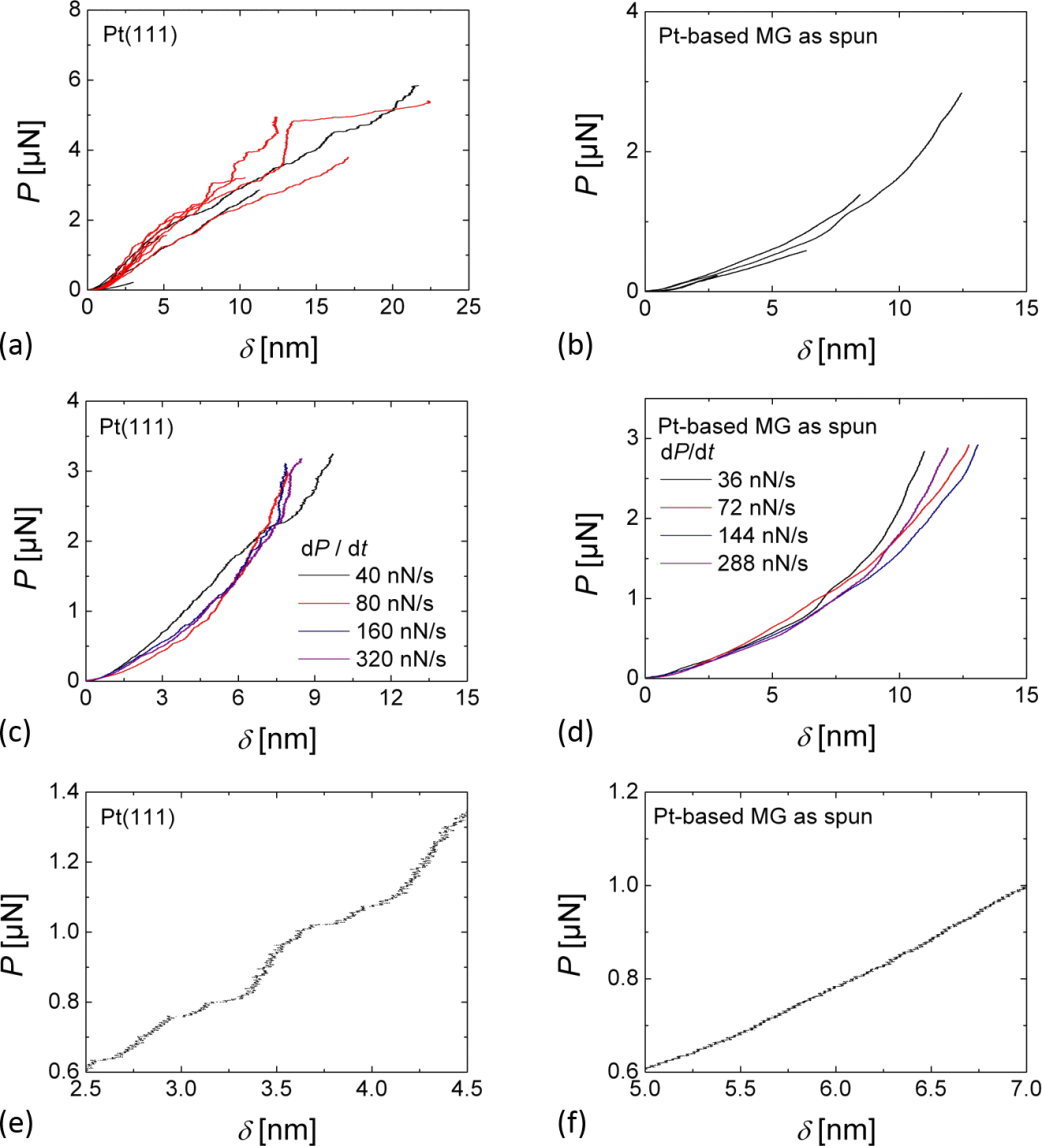
*P*–δ curves as a function of (a,b) the maximal load *P*_max_ and (c,d) the loading-rate on (a,c) Pt(111) and (b,d) Pt_57.5_Cu_14.7_Ni_5.3_P_22.5_ metallic glass; in (e,f) magnifications of *P*–δ curves at the plasticity on-set are shown for Pt(111) and Pt_57.5_Cu_14.7_Ni_5.3_P_22.5_ metallic glass, respectively.

Figure 7a and Figure 7b shows indentation curves for Pt(111) and Pt_57.5_Cu_14.7_Ni_5.3_P_22.5_ metallic glass for different values of *P*_max_. For Pt(111), the *P*–δ curves from different series of measurements overlap with each other, demonstrating the good reproducibility of the method. The *P*–δ curves on the crystalline Pt(111) show clear pop-ins. In the low load part of indentation curves (*P* = 0.6–1.4 µN) the pop-ins have a length of the order of 1 Å and are attributed to the activation of single dislocations. The load corresponding to the first pop-in event was found to scatter from *P*_y_ = 186–633 nN at an indentation depth δ_y_ = 1.43–2.54 nm. With increasing load we observed an increase of the length of the pop-ins up to several nanometers, which corresponds to the simultaneous activation of several dislocations. For the metallic glass, the *P*–δ curves measured with different *P*_max_ values also overlap well. In contrast to the serrated flow of the crystalline Pt(111) evidenced by the occurrence of pop-ins, the plastic flow of the metallic glass is found to be continuous as demonstrated by the absence of pop-ins in the *P*–δ curves. Figure 7b and Figure 7d show indentation curves on Pt(111) and Pt_57.5_Cu_14.7_Ni_5.3_P_22.5_ metallic glass for varying loading rates. For both samples no loading-rate dependence could be observed: the *P*–δ curves are found to overlap eachother.

**Figure 8:**
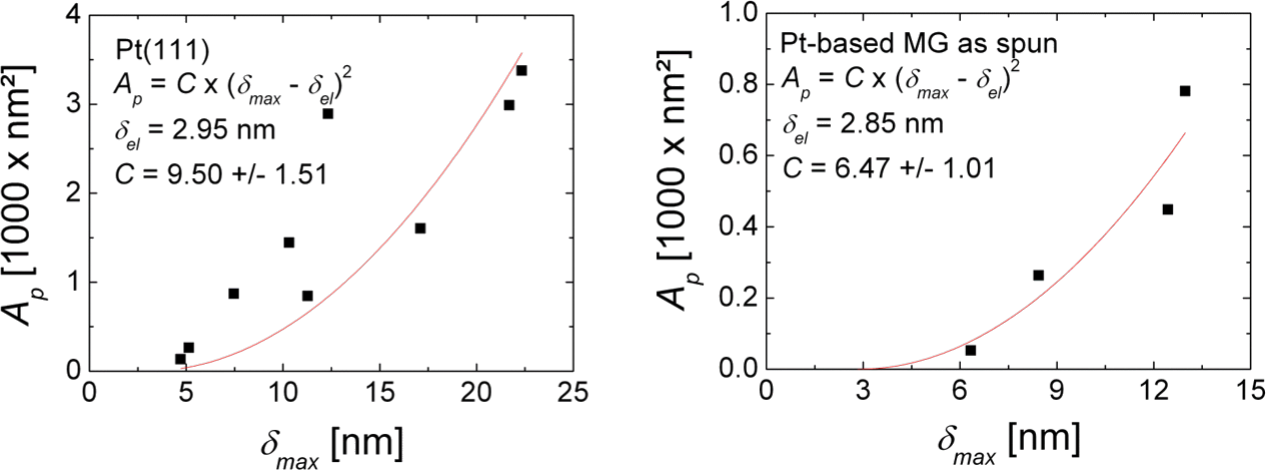
Projected area *A*_p_ as determined from nc-AFM images as a function of the maximal indentation depth δ_max_ as calculated from force–distance curves for (a) Pt(111) and (b) Pt_57.5_Cu_14.7_Ni_5.3_P_22.5_ metallic glass.

Figure 8 shows the projected area *A*_p_ as a function of the maximal indentation depth δ_max_ for both samples. In order to evaluate the geometry of the indenter the data points for both samples were fitted with a parabolic function of the type *A*_p_ = C × (δ_max_ – δ_el_)^2^, where δ_el_ is the indentation depth at the elastic limit and *C* is geometric factor that depends on the opening angle of the indenter. In this work δ_el_ was set as the maximal indentation depth corresponding to the smallest load at which a remaining indent was observed. For Pt(111) δ_el_ = 2.95 nm and for Pt_57.5_Cu_14.7_Ni_5.3_P_22.5_ metallic glass δ_el_ = 2.85 nm. Given the triangular shape of the indent obtained by AFM indentation the geometric factor *C* was set in analogy to a Berkovich indenter as 
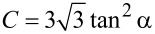
, where α is the half-opening angle of the indenter. This analogy is supported by the assumption that in our AFM measurements the indentation proceeded from the sharp corner of a single diamond nano-crystallite. From our results on Pt(111) we find *C* = 9.50 corresponding to α = 55.9° and for Pt_57.5_Cu_14.7_Ni_5.3_P_22.5_ metallic glass we obtain *C* = 6.47 corresponding to α = 50.7°. These results are reasonably close each other but deviate from the value of the half-opening angle of the reconstructed tip shown in Figure 3 of α = 74°. As stated above the projected area determined from nc-AFM images of indents is underestimated due to tip-convolution effects and results in an underestimated half-opening angle of the indenter.

## Discussion

A single diamond-coated AFM tip was used to perform several series of indentations at various maximal loads and loading rates on Pt(111) and a Pt_57.5_Cu_14.7_Ni_5.3_P_22.5_ metallic glass. Throughout our experimental series results have been highly reproducible. We find very similar shape of indents and their pile ups in nc-AFM images and an excellent overlap of the corresponding *P*–δ curves. Unlike in nanoindentation experiments with a standardized and macroscopically well-defined indenter geometry (such as a Berkovich tip), the indenter used in our AFM experiments is a diamond crystallite which is part of the diamond coating of the AFM tip. The geometry of this microscopic indenter was reconstructed from nc-AFM images selected from different experiment series on both samples recorded several months apart. Though the shape of imaged indents and of the pile-up geometry on both samples differed significantly, the reconstructed tip shape is very similar. In all cases the reconstructed tip shape is a three-sided pyramid. The angle of each side with regard to the base of the pyramid is found to be about 16°, while its half opening angle is about 74°, which may represent the corner of a single diamond nano-crystallite. We also determined the half-opening angle α of our indenter from the depth dependence of the projected area of indents and obtained α = 50–55°. As mentioned above the difference in the α values can be explained by the underestimation of the projected area from nc-AFM images of indents due to tip-convolution effects.

The stability of the microscopic indenter shape throughout our experimental study allows us to compare the nanometer-scale plastic flow of Pt(111) and Pt_57.5_Cu_14.7_Ni_5.3_P_22.5_ metallic glass. Our AFM indentation results reveal fundamentally different deformation mechanisms. For the crystalline Pt(111) the plastic deformation is accommodated over much larger distance than for the metallic glass. In our study the AFM indentation of Pt(111) is similar to KBr(100) or Cu(100) where plastic flow has been found to extend over several 100 nm from the AFM indentation site [7–8]. In contrast, the plastic flow of Pt_57.5_Cu_14.7_Ni_5.3_P_22.5_ metallic glass is highly concentrated around the AFM indenter.

In a recent study, we have compared the nano-scale wear of Pt(111) and Pt_57.5_Cu_14.7_Ni_5.3_P_22.5_ metallic glass by AFM scratching in UHV. The friction forces measured during reciprocal scratching with a diamond-coated silicon tip were found to be four times higher for the metallic glass than for Pt(111). This difference has been explained in [13] on the basis of the respective deformation mechanisms of crystalline and amorphous metals, namely easy dislocation mobility for Pt(111) and high resistance to plastic flow for Pt_57.5_Cu_14.7_Ni_5.3_P_22.5_ metallic glass. The latter was found to be mediated by thin shear bands at moderate loads of *P* in the range of 400 to 1.5 µN until the sliding contact merged in a single shear zone at higher load. Note that the high shear rate parallel to the surface in those scratching experiments as compared to the slower indentation experiments presented here is likely responsible for the occurrence of shear bands that can abruptly relax elastic stresses.

The nanometer-scale plastic flow of Pt(111) occurs by discrete events, i.e., pop-ins that correspond to the activation of dislocations. In the low-load regime the length of pop-ins is a few angstroms and probably corresponds to the activation of single dislocations. At higher loads, *P* > 3 µN, the pop-in length increases up to several nanometers, which corresponds to the simultaneous activation of several dislocations. These findings are again in good agreement with previous AFM indentation results on crystalline KBr(100) [7] and Cu(100) [8]. The increased pop-in length at higher loads is also in good agreement with nanoindentation results on (111)-oriented fcc-metal surfaces such as Au(111), for which burst-like dislocations activation has been observed [3]. However, the nanometer-scale plastic flow of Pt_57.5_Cu_14.7_Ni_5.3_P_22.5_ metallic glass is found to be continuous, without any signs of discontinuous events such as pop-ins. The observation of continuous plastic flow during AFM indentation on the metallic glass is in contrast to the observation of serrated flow in nanoindentation experiments on metallic glasses, where the occurrence of pop-ins has been associated to the generation and propagation of shear bands [4]. In nanoindentation experiments the plastically deformed volume is in the range of several cubic micrometers. Plastic deformation is mediated by the cooperative activation of several shear transformations zones (STZs) [21] and is often characterized by a serrated flow. Single pop-ins in nanoindentation load–displacement curves correspond then to the operation of single shear bands. In the following we introduce previous studies on homogeneous flow in very small metallic glass samples and relate them to our results. We will then discuss alternative concepts leading to homogeneous flow and finally the role of strain rates.

The investigation of the plastic flow of micro- and nano-fabricated test samples prepared from metallic glasses by focus ion beam (FIB) with volumes in the range of a few cubic micrometers to 0.05 µm^3^ has revealed a transition from localized to delocalized plastic deformation upon reducing the sample size down to a diameter of the order of 100 nm [9]. For micrometer-sized samples studied in compression, plastic flow is characterized by visible shear steps at the sample surface and discrete load drops in the stress–strain curves that are attributed to the operation of single shear bands. Below a critical sample diameter though, the surface of samples tested in compression or tension are devoid of shear steps or shear bands and the plastic deformation has in this case been consequently described as homogeneous [10]. In [10] the size-induced transition from serrated to homogeneous plastic deformation has been discussed on the basis of an energetic argument, where below a critical sample size the elastic energy stored in the sample can no longer account for energy release to the area of a shear band traversing the sample. More recently, it has been shown that despite the disappearance of shear bands and shear steps at sample surfaces upon the decrease of sample size one could still observe intermittence in stress–strain curves [11–12]. The magnitude of plasticity events was much reduced and their frequency higher than for larger samples. This effect was attributed to local shear transformation events [12] that could not be resolved in previous works, probably due to limitations in the force and displacement resolution of nanoindentation. From these previously reported results it can be inferred that plastic deformation of metallic glasses becomes rather delocalized than homogeneous below a critical volume of ca. 0.05 µm^3^ but does not lose its intermittent character. In the experimental results presented in this study, force and displacement resolution are higher than in any of the studies discussed above, and we still observe no serrated but only homogeneous flow.

The generation of a shear band has been discussed to require the storage of a sufficient amount of elastic energy within the strained volume to overcome the interfacial energy of a shear band [10,12]. In our case the elastic energy stored in the contact corresponds to 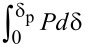 where δ_p_ is the penetration depth at the onset of plastic deformation that can be taken as the δ_max_ value for *P*_max_ = 0.6 µN (see Figure 2). The shear band energy can be calculated according to 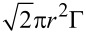, where *r* is the radius of the plastic zone and Γ ≈ 10 J/m² is a higher bound for the energy per unit area of the shear band [10]. The critical shear band size can be equated as


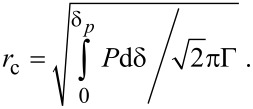


For our measurement with *P_max_* = 0.6 µN we find 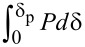 = 1.61 × 10^−15^ J and *r*_c_ ≈ 6 nm. From Figure 4 the size of the plasticity zone can be estimated to be *r*_p_ ≈ 5 nm by measuring the distance between pile-up and the center of the indent. The value estimated for *r*_p_ is lower than for *r*_c_, inferring that in our AFM indentation experiments the generation of shear bands was not energetically favorable. Instead we observe that the plastic deformation of Pt_57.5_Cu_14.7_Ni_5.3_P_22.5_ metallic glass at the nanometer-scale occurs by a continuous material flow around the tip. The contrast between serrated and homogeneous flow for Pt(111) and Pt_57.5_Cu_14.7_Ni_5.3_P_22.5_ metallic glass is confirmed by the different characteristics of the remaining indents on both samples. Plastic deformation of Pt(111) was accommodated by dislocations, over larger distance than in the case of Pt_57.5_Cu_14.7_Ni_5.3_P_22.5_ metallic glass. The pile-up around indents in Pt_57.5_Cu_14.7_Ni_5.3_P_22.5_ metallic glass are also devoid of shear steps in contrast to nanoindentation experiments [22].

The difference in the mechanisms involved in the nanometer-scale plastic deformation of Pt(111) and Pt_57.5_Cu_14.7_Ni_5.3_P_22.5_ metallic glass is also reflected in the hardness. Although the *H* values determined from AFM indentation experiments are overestimated due to the underestimation of the projected area it is interesting to note that the hardness ratios for Pt_57.5_Cu_14.7_Ni_5.3_P_22.5_ metallic glass and Pt(111) are slightly higher from AFM indentation than from nanoindentation. The values obtained from our measurements were:


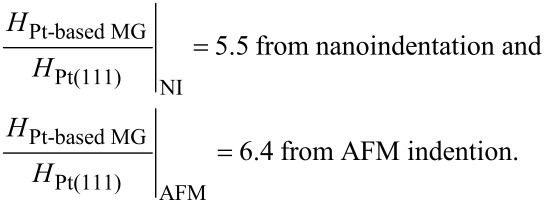


This may point at different size effects for the plasticity of Pt(111) and Pt_57.5_Cu_14.7_Ni_5.3_P_22.5_ metallic glass. For crystalline materials the indentation size effect has been rationalized on the basis of geometrically necessary dislocations with Burgers vectors normal to the plane of the surface [23]. In this case the hardness decreases with the indentation depth according to 
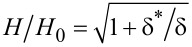
, where *H*_0_ is the hardness in the limit of infinite depth and δ^*^ is a characteristic length depending on the indenter geometry, the shear modulus, the Burgers vector, and *H*_0_. For metallic glasses an indentation size effect has also been observed and has been discussed on the basis of accumulation of STZs during indentation and subsequent shear softening at larger indentation depths resulting in an increase of the hardness at smaller indentation depth [24].

In order to shed light on our AFM indentation results at different loading rates, we discuss previous observations on the effect of loading rate and homologous temperature on the plastic flow of metallic glasses during nanoindentation. At higher loading rates and homologous temperatures, the serrated character of plastic flow recorded on metallic glasses during nanoindentation disappears [4,25]. The effect of the strain rate on the transition from serrated to homogeneous plastic flow of metallic glasses has been explained by the idea that a single shear band cannot accommodate the imposed strain rapidly enough at high strain rates [4]. According to the authors, at high strain rates the applied strain is instead accommodated by the simultaneous operation of multiple shear banding events that can no longer be distinguished in load–displacement curves. Further, the effect of temperature on the transition from serrated to homogeneous plastic flow of metallic glasses has been discussed in [25] on the basis of the thermal activation of STZs [21]. On macroscopic scale, the flow stress in metallic glasses or activation of STZs has been reported to depend on the strain rate [26]. This has also been observed by nanoindentation where at higher rates the hardness increases [27] and has been used to determine the size of STZs.

In our AFM indentation experiments no rate dependence could be observed on Pt(111) or Pt_57.5_Cu_14.7_Ni_5.3_P_22.5_ metallic glass. Our observation of no loading-rate dependence in Pt(111) is in good agreement with the absence of strain-rate sensitivity of coarse crystalline fcc metals at low homologous temperatures. The absence of loading-rate further emphasize that during our AFM indentation experiments on Pt_57.5_Cu_14.7_Ni_5.3_P_22.5_ metallic glass plasticity is not mediated by shear banding or the activation of STZs but involves alternative mechanisms. Our experiments on Pt_57.5_Cu_14.7_Ni_5.3_P_22.5_ metallic glass were performed at a homologous temperature *T*_H_ = 0.6, which is far below the reported value for the transition from serrated to non-serrated flow of metallic glasses during nanoindentation [26]. For Pt_57.5_Cu_14.7_Ni_5.3_P_22.5_ metallic glass we claim that the volume that is plastically deformed during AFM indentation is not large enough to yield a significant accumulation of STZs and their autocatalytic multiplication, which would result in shear softening.

## Conclusion

AFM indentation was used to quantitatively and reproducibly determine the hardness and deformation mechanisms of Pt(111) and a Pt_57.5_Cu_14.7_Ni_5.3_P_22.5_ metallic glass with unprecedented resolution in imaging and force curves thanks to operation and sample preparation in ultra-high vacuum. At the nanometer-scale the plastic deformation mechanisms of Pt(111) remain consistent with the serrated mechanisms operating at larger scale: Plasticity is accommodated over large distances by dislocation gliding and no rate dependence is observed. For Pt_57.5_Cu_14.7_Ni_5.3_P_22.5_ metallic glass the nanometer-scale plastic deformation is rate independent, continuous and localized around the indenter, which contrasts with the observation of serrated flow at the micrometer-scale and the rate dependence of flow stress of metallic glasses at larger scales. The results demonstrate a lower size limit for metallic glasses below which shear transformation mechanisms are not activated by indentation. In the case of metallic glass, we conclude that the energy stored in the stressed volume during nanometer-scale indentation is insufficient to account for the interfacial energy of a shear band in the glassy matrix.
